# Magnetic Resonance Sialography Findings of Submandibular Ducts Imaging

**DOI:** 10.1155/2013/417052

**Published:** 2013-07-25

**Authors:** Nezahat Karaca Erdoğan, Canan Altay, Nesibe Özenler, Tuğba Bozkurt, Engin Uluç, Berna Dirim Mete, İsmail Özdemir

**Affiliations:** ^1^Department of Radiology, Izmir Atatürk Research and Training Hospital, Basın Sitesi, Karabağlar, 35360 Izmir, Turkey; ^2^Department of Radiology, Medical School, Dokuz Eylul University, Inciralti, 35340 Izmir, Turkey; ^3^Department of Radiology, Balıkesir Atatürk State Hospital, Yıldız Mahallesi Soma Caddesi No. 1, 10100 Balıkesir, Turkey; ^4^Universal Ege Health Hospital, 35220 Izmir, Turkey

## Abstract

*Purpose*. We aimed to assess the problem solving capability of magnetic resonance sialography (MR sialography), a noninvasive method for imaging submandibular gland ducts and determining duct-related pathologies, by comparing diseased and healthy cases. *Materials and Methods*. We conducted radiological assessment on a total of 60 submandibular glands (mean age 44.7) in 20 cases and 10 volunteers. MR sialography examinations were conducted with single-shot fast spin-echo sequence by using a surface coil placed on the submandibular gland. Each gland was evaluated in terms of the length, width and stricture of the main duct, as well as the difference between the intraparenchymal duct width, and the main duct width. Statistical analysis was performed. *Results*. In the MR sialography the primary duct mean length was determined as 51 mm (40–57 mm) in all submandibular glands. On the MR sialography imaging, the visualization ratio of the ductal system of submandibular gland was evaluated in the cases and volunteers. *Conclusion*. MR sialography is an effective and a noninvasive method in imaging submandibular gland ducts, demonstrating the presence, location and degree of stricture/dilatation, and elucidating the disease etiology.

## 1. Introduction

Conventional and digital sialography, US, CT imaging, and MR sialography (MR-Si) methods are used in the assessment of major salivary gland ducts [1, 2]. In the imaging of the primary submandibular gland duct and intraglandular branches, conventional sialography is the golden standard technique [3]. However, sialographic examination is an invasive method which requires the use of an iodized contrast agent and causes ionizing radiation exposure. Its use is contraindicated in cases with iodine allergy, thyroid gland disease, or active infection [4, 5]. The primary limitation of conventional sialography is that stricture cannot be overcome at most times in cases with duct obstruction and therefore examination cannot be carried out, and even if the examination is performed, the proximal segment of the stricture of the submandibular duct cannot be evaluated.

The most prevalent pathology of submandibular gland ducts is calculus and calculus-induced inflammations [6]. 80–82% of all salivary gland calculi are traced in submandibular ducts [6, 7]. 

In recent years, conventional sialography has been replaced by MR-Si examinations in which the patient's own salivary secretion is used as a natural contrast agent and which can be conducted quickly and without any complications [6, 8–10]. In this study, we aimed to assess the problem solving capability of MR-Si, a noninvasive method for imaging submandibular gland ducts and determining duct-related pathologies, by comparing diseased and healthy cases.

## 2. Materials and Methods

In our study, we conducted radiological assessment on a total of 60 submandibular glands, 40 submandibular glands of 20 cases (13 females and 7 males) between 18 and 71 years of age (mean age 42.9) who applied to our hospital between September 2007 and July 2011 with complaints of pain and swelling in submandibular glands, and 20 submandibular glands of 10 volunteers (6 females and 4 males) between 17 and 38 years of age (mean age 26.1). No statistical comparison was performed in the age groups between cases and volunteers. All patients were referred from the otolaryngology outpatient clinic for MR-Si with presenting symptoms of intermittent pain and swelling of the submandibular glands related to eating (45%), persistent swelling (38%), and chronic sialadenitis (16%). Persistent bilateral swelling was present in two patients. Twenty submandibular glands were clinically normal and thirteen of them found normal ultrasonographic findings in disease group. The normal sides of the disease group were not included into the control group in this study. Conventional sialography was not performed in any of the disease group cases. 

The study was approved by the review board of our department. All patients gave informed consent to participate before beginning the study.

The cases were first subjected to MRI with conventional sequences and then to MR-Si examination by stimulating their salivary secretion with lemon. 

### 2.1. Conventional MRI

In MRI, fast spin echo (FSE) T1-weighted axial images (TR: 400 msec, TE: 17, number of signal acquisitions (NSA): 3, matrix: 256 × 256, FOV: 250, section thickness: 4 mm, intersection gap: 1 mm, and examination period: 3 min 4 sec), fat-suppressed FSE T2-weighted axial images (TR: 2500, TE: 110, NSA: 2, echo train length: 15, matrix: 256 × 256, FOV: 250, section thickness: 4 mm, intersection gap: 1 mm, and examination period: 3 min 16 sec), and FSE T2-weighted images in the coronal plane (TR: 4872, TE: 100, NSA: 2, echo train length: 15, matrix: 256 × 256, FOV: 250, section thickness: 4 mm, intersection gap: 1 mm, and examination period: 1 min 57 sec) were obtained by 1.5 T scanner (Gyroscan Intera, Philips Medical Systems, Best, The Netherlands) in the maxillofacial area by using a 20 cm surface coil (flex M coil). Fat saturation was suppressed the signals from the subcutaneous fat. Intravenous paramagnetic agent was not used in the examination, since it could obscure the signal changes in the gland parenchyma. The size of submandibular glands, signal properties in T1- and T2-weighted axial images, duct content, and the presence of mass were evaluated in the conventional MRI. 

### 2.2. MR Sialography

Each patient underwent MR-Si using T2-weighted sequences. MR-Si examinations were conducted with 2D single-shot fast spin-echo (SSFSE) sequence (TR/TE/NSA: 8000/800/5, matrix: 192 × 256, FOV: 110 × 110 mm, bandwidth: 32 kHz, TSE factor 54, spectral presaturation with inversion recovery (SPIR), and examination period: 2 min 20 sec) with parallel imaging (SENSE) by using surface coil (synergy flex M) placed on the submandibular gland. The slice thickness was 3-4 cm, depending on the submandibular gland volume. Gadolinium-containing contrast agent was not administered. 2D SSFSE sequences were acquired in a sagittal-oblique plane parallel to the Wharton duct at 2–4 different angular planes to display the submandibular gland and ductal system. Patients were hydrated for 2-3 days prior to the examination in order to increase salivary secretion and image quality. Before the examination, lemon juice (5 cc) was given to all patients to increase the salivary secretion. Patients, who have parotitis or Stensen's duct obstruction, are very sensitive to the lemon juice which stimulates salivation and may cause pain. Prior to investigation, patients should be questioned for the parotid gland disease.

**Figure 1:**
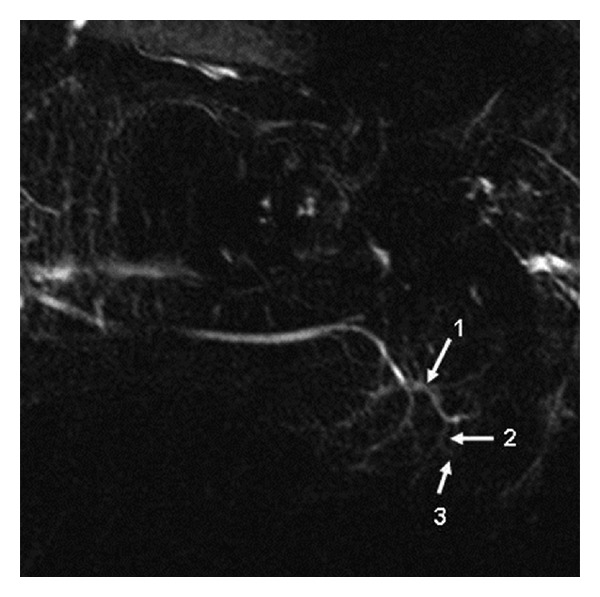
Submandibular gland ducts by MR sialography. 1: primary, 2: secondary, and 3: tertiary branches.

The morphology of submandibular gland ducts and the presence of accessory ductus were evaluated in the MR-Si images. Intraparenchymal ducts were classified as primary, secondary, and tertiary branches for terminological convenience and ease of identification (Figure 1) [1, 9]. Each gland was evaluated in terms of the length, width, and stricture of the primary duct, as well as the difference between the intraparenchymal duct width and the primary duct width. 

Diameter measurements were obtained as follows. First, sagittal-oblique 2D SSFSE images at 2–4 different angular planes were reviewed, and the one with a well-demonstrated ductal system of submandibular gland with the least superimposed artifacts was used for measurements. This was done by drawing a line along the center of the primary duct as viewed on the MR-SI image. This was the long-axis view of the primary duct. The assessment of the primary ductal length of the submandibular gland was performed between the level of submandibular gland edge and sublingual caruncle. The long-axis diameter of primary duct was measured using the electronic caliper provided by the picture archiving and communications system (Angora PACS, Datamed Medical Systems, Ankara, Turkey). This allowed all anteroposterior diameter measurements to be obtained in a plane perpendicular to the long axis of the primary duct (Figure 1). The inner anteroposterior diameters of the primary duct of submandibular gland were measured at four locations at 10 mm intervals, starting from the origin of the primary duct at the edge of the submandibular gland (level 1) to a point of the ostium at the sublingual caruncle (level 4). Each measurement was performed three times, and the mean values were recorded. one mm was considered the upper limit of normal values for the diameter of the primary duct of submandibular gland [10]. The inner diameters of the primary branch were obtained with the same method between the origin point from primary duct and the level of ramification of the primary branches. The relationship between primary duct and branch width in patients with stone and sialoadenitis and normal volunteers was studied using independent samples *t*-test for independent samples. In our study, the longitudinal diameter measurements on primary, secondary, and tertiary branches of submandibular gland were not performed.

## 3. Results

In our study, a total of 60 submandibular glands (40 submandibular glands of 20 patients and 20 submandibular glands of 10 volunteers without any clinical complaints) were assessed initially by MRI and then by MR-Si examination. 

### 3.1. Conventional MRI

In the MRI of 20 submandibular glands obtained from the volunteer group, submandibular gland dimensions, parenchyma signal, and homogeneity were found to be normal.

In the MRI of 40 (*n* = 40) submandibular glands obtained from the diseased cases, parenchymal heterogeneity and extension in intraparenchymal ducts (*n* = 11), atrophy and fat infiltration (*n* = 4), mass lesion next to the posterior of the submandibular gland (*n* = 1), findings of stone and obstruction in the main distal of the duct (*n* = 5), and focal segmental dilatations in the intraparenchymal ducts (*n* = 4) were detected (Table 1). In the rest of the group (*n* = 15), MRI findings of the submandibular gland were observed to be normal.

### 3.2. MR Sialography

**Figure 2:**
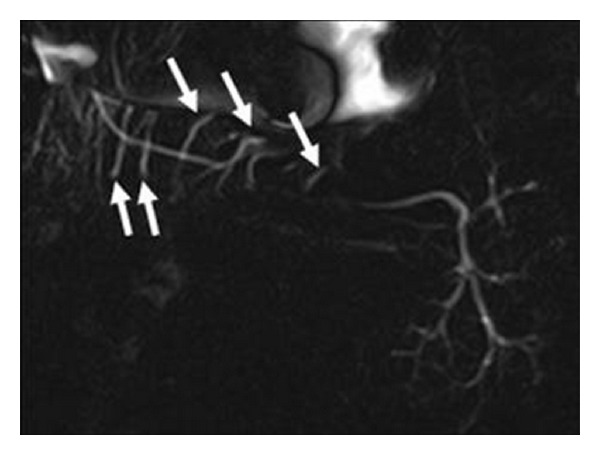
Ducts of Rivinus crossing the main submandibular gland duct vertically (arrows) in MR sialography examination.

In the MR-Si examination of all submandibular glands in this study, the primary duct could be monitored in 54 of 60 glands (90%), and primary branches could be monitored in all glands (100%), secondary branches could be monitored in 54 of 60 glands (90%), tertiary branches could be monitored in 48 of the 60 glands (80%) (Table 2). In 9 of the examined submandibular glands, 1 to 8 ducts of Rivinus belonging to sublingual glands were visualized; these ducts, most of which crossed the primary duct vertically, measured 10–20 mm in length, stretching from the middle of the primary duct to the distal (Figure 2). 

Diameter and length measurements were not performed in a gland in the control group, because the primary duct could not be monitored. The lumen diameter of the other measurable main submandibular ducts and intraparenchymal branches was found to be below 1 mm in all volunteers. The length of the primary duct varied between 40 mm and 57 mm, and the mean length was determined as 51 mm.

**Figure 3:**
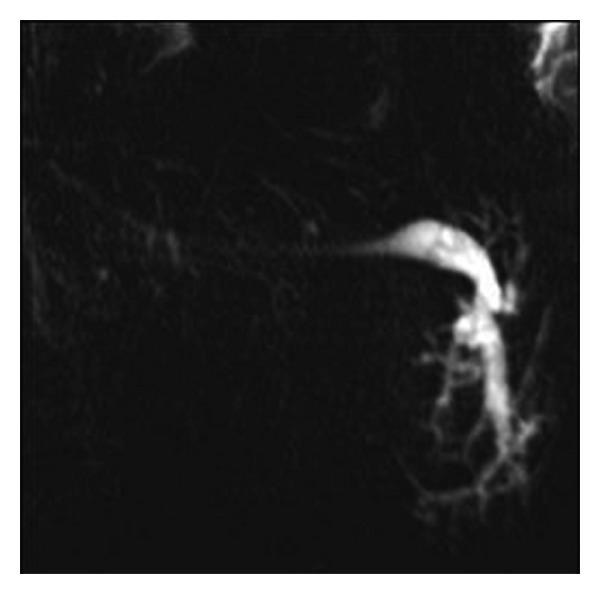
Dilatation and stricture in the ducts of submandibular gland consistent with chronic sialadenitis on MR sialography images.

**Figure 4:**
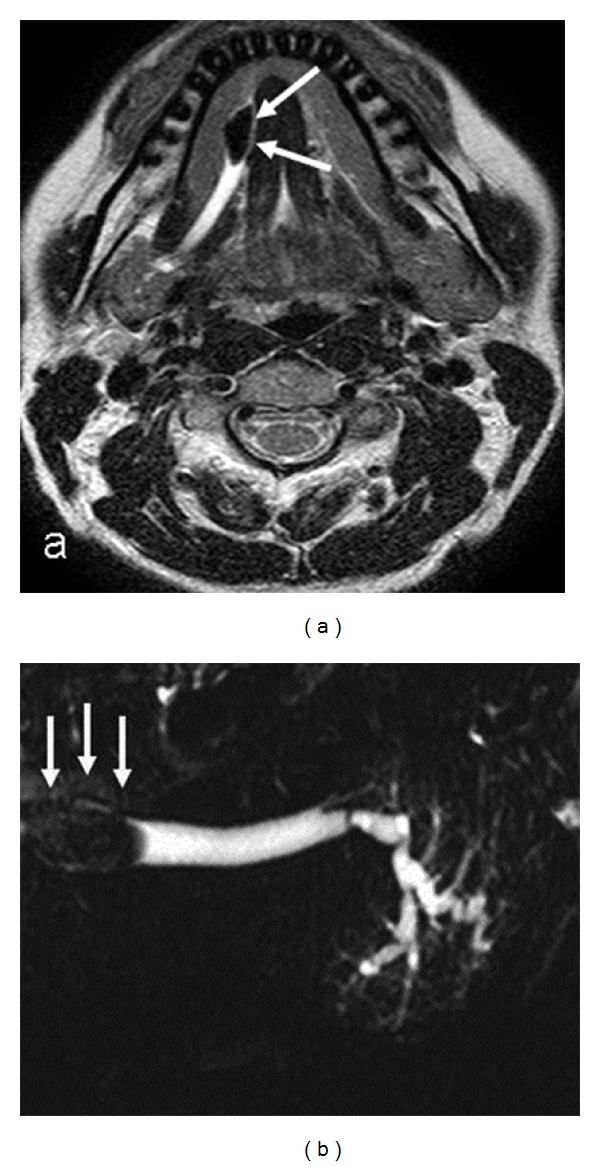
Axial T2-weighted MR image (a) and MR sialography (b) images show the stone located in the right submandibular gland main distal of the duct.

**Figure 5:**
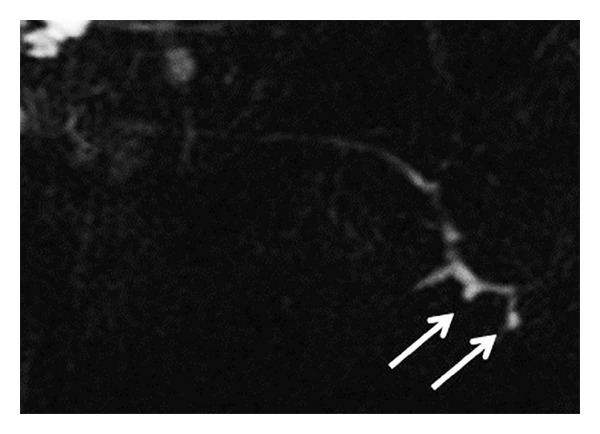
Amputation in the secondary branches of the left submandibular gland in the case with Sjögren's syndrome, pruned tree appearance (arrows) on MR sialography images.

Accessory ducts were detected in 6 of the 40 glands (15%) in the group with clinical complaints (2 opening to the main proximal of the duct, 2 opening to the middle section, and 2 opening to the distal). The findings include contour disorder concordant with the chronic sialadenitis in the primary duct and segmental stricture/dilatation in the intraparenchymal ducts (*n* = 8) (Figure 3), segmental dilatation in the inner gland ducts (*n* = 7), and stone and obstruction in the primary duct (*n* = 10) (Figures 4(a) and 4(b)). In our study, we detected stones in the ducts of 10 glands—a single stone in 7 glands and multiple stones in 3 glands. We monitored 4 stones in the right primary duct and two stones in the left primary duct in one case and 4 stones in the primary duct in the other case. The size of the stones varied between 4 and 14 mm, mean size: 4.8 ± 1.3 mm, and 65% were located in or next to the orifice of the primary duct. One case (*n* = 1) revealed displacement, parallel positioning, and distortion in primary and secondary branches caused by the pressure exerted on intraparenchymal duct structures by the submandibular fossa localization of the mass (Table 3). The image of a pruned tree was observed in the secondary branches of both submandibular glands of a case followed up due to renal tubular acidosis and later diagnosed with Sjögren's syndrome based on radiological findings (*n* = 2) (Figure 5). The MR-Si examination for the left parotid of the same case revealed cystic duct dilatation and “apple tree appearance” caused by the increase in gland.

The primary duct length of 20 cases with clinical complaints varied between 38 mm and 62 mm, and the mean length was found as 50 mm.

The primary duct calibration of 10 glands with stones was 1.3–6.3 mm (mean 3.5 mm), and the primary branch calibration was 0.9–4.1 mm (mean 2.4 mm), while the primary duct calibration was 0.9–5.1 mm (mean 2.2 mm), and primary branch calibration was 0.6–3.5 mm (mean 2.1 mm) in 8 cases with chronic sialadenitis. The primary duct calibration was found to be 0.6–3.1 mm (mean 1.5 mm) in 7 glands with only intraparenchymal ductal ectasia, and in the same group, the primary branch width was detected and 0.6–1.4 mm (mean 0.9 mm). The primary duct calibrations and primary branch calibration of 13 normal glands in cases groups were found to be 0.6–2.0 mm (mean 1 mm) and 0.5–1.3 mm (mean 0.8 mm), respectively. The difference between the primary duct width in volunteers and patients with stones was significant (*P* value: 0.035). However, there was no significant difference between the primary duct width in volunteers and patients with sialoadenitis (*P* value >0.05) and primary branch width in all groups (*P* value >0.05).

## 4. Discussion

MR-SI, in which the patient's salivary secretion is used as contrast agent, is a method of examination based on a basic principle of using T2-weighted sequence for monitoring liquids [10–12]. MR-Si is not only a quick method, but it is also easily applicable to every case which does not show contraindications in MRI. Its superior properties also include its capability of displaying the duct diameter in its actual value due to the nonuse of the contrast agent (administration of contrast agent increases lumen pressure and makes the diameter look wider than it actually is) as well as displaying the ducts of Rivinus.

In a comparative study in the literature, in which different sequences were used to evaluate submandibular ducts, the most successful results were obtained with 3D CISS sequence for showing the main, primary, and secondary (100%) branches; however, tertiary branches were not displayed in this comparative study. Also, RARE (40%) and TSE (5%) sequences employed in this study failed to show the secondary branches [1]. In a different study carried out with head-neck coil, 10% success was achieved in MR-Si in terms of the imaging of tertiary branches in the volunteer group [9]. Examination with surface coil is preferred in recent years due to the insufficient spatial resolution of MR-Si images obtained with head-neck coil, its restriction in displaying secondary and tertiary branches, and its low signal-to-noise ratio [1, 6, 9, 11, 13]. In our study, the success of imaging submandibular gland ducts was 90% in the main duct, 100% in primary branches, 90% in secondary branches, and 80% in tertiary branches. Because we utilized saliva as a natural contrast material in this study, visualization of the glandular ductal system was increased by stimulating salivation with lemon juice. During the MR examination performed, the decrease of the TSE factor and increase of the NSA might attribute to the improvement for the SN ratio. We believe that the use of surface coil contributed to our success in imaging secondary and tertiary branches, and that the impairment of the primary duct angle due to patient mobility rather than technical reasons was responsible for the failure of obtaining good images in some cases.

In Wharton's duct, sublingual glands lined up along the sublingual plica on both sides of the tongue frenulum open separately into the oral cavity via 10–20 ducts of Rivinus [14]. In our study, we could achieve 50% success in the patients group and 90% success in the healthy group in imaging 2–8 ducts of Rivinus crossing vertically the middle and distal sections of Wharton's duct.

In conventional sialographies, the administration of the contrast agent manually in different amounts and pressures leads to the dilatation of central duct structures or to different measurements in duct diameters [15]. The main submandibular duct diameter was found to be 1–3 mm in conventional sialographic examinations, whereas Wharton's duct diameter was measured to be below 1 mm in the MR-Si studies carried out by Becker et al. by using EXPRESS sequence [1, 10]. In the present study, we determined the width of the primary duct and primary branches as 1 mm and below in the normal control group and the healthy glands constituting the patients group. We believe that these MR-Si values obtained without the exertion of pressure and administration of contrast agent in the lumen have provided more reliable and factual results.

The length of the main submandibular duct is approximately 50–60 mm [1]. In our cases, this value was determined as 40–57 mm in the control group (mean 51 mm) and 38–62 mm in the group with clinical complaints (mean 50 mm). This value was evaluated to be consistent with the 50–60 mm length specified in the literature [1].

The most commonly observed causes of chronic sialadenitis are bacterial infections; they spread from the oral cavity in an ascending way due to the frequently decreasing salivary flow. In addition, secondary infections are induced also by the partial obstruction of the duct by mucus spill, debris, and stones [7, 16]. Chronic sialadenitis is recognized in MR-Si by segmental dilatation and stricture in the ducts [2]. In our study, segmental dilatation and stricture were observed in the ducts of 8 glands which were diagnosed with chronic sialadenitis. In these patients, primary duct width was significantly wider than that of the volunteers in our study. Probably, this situation is associated with chronic and recurrent inflammation of the mucosal surface of the submandibular primary duct. Recurrent inflammations cause damage in the tissue repair mechanism of duct wall. As a result, irreversible ductal dilatation occurs. However, duct diameters were found to be at normal limits (below 1 mm) in 3 of the cases. Hence, we concluded that segmental dilatation and stricture in the duct (occasionally on a relative level) have more diagnostic value than the dilatation of the duct diameter in chronic sialadenitis cases. 

The limitations of MR-Si examination include the facts that it cannot be applied in individuals with pacemaker as well as in claustrophobic individuals and that the image is affected by patient mobility and the loss in saliva amount [1, 5, 6]. Disadvantages of MR-Si include overall MR imaging contraindications; misregistrations artifacts caused by dental amalgam may poor imagination of stone or stenosis near the main ductus of submandibular gland [10]. However, dental amalgam did was observed in our study. Nevertheless, MR-Si has some superiorities over the conventional sialography: it can be used in individuals with iodine allergy and it does not contain ionized radiation; it is a reliable method in acute infections; it is used in cases in which no success is achieved by conventional sialography; it is the only modality displaying both the gland function and the proximal ductal structures in obstructions [5, 6]. 

In conclusion, MR-Si is a noninvasive method of imaging the ductal system of submandibular gland and parotid gland. This MR technique has the potential to provide a comprehensive examination for the detailed anatomic assessment of the major salivatory glands. We believe that MR-Si will be attributed greater significance in the future and will contribute to diagnosis in routine use, as it is the only modality that can show the actual lumen width of the duct due to the nonuse of induct contrast agent and display the ducts of Rivinus and duct pathologies.

## Figures and Tables

**Table 1:** Submandibular gland conventional MRI findings.

Conventional MRI findings in cases with clinical complaints (submandibular gland *n* = 40)	
Parenchymal heterogeneity	11
Atrophy	4
Mass	1
Stone	5
Ectasia	4
Normal	15

**Table 2:** Visualization rates of submandibular ducts by MR Sialography in patient and control group cases.

Ratio of visualisation of the submandibular ductus in MR sialography			
	Diseased cases (*n* = 40)	Control groups (*n* = 20)	Totally (*n* = 60)
Primary duct	36 (90%)	18 (90%)	54 (90%)
Primary branches	40 (100%)	20 (100%)	60 (100%)
Secondary branches	36 (90%)	18 (90%)	54 (90%)
Tertiary branches	32 (80%)	16 (80%)	48 (80%)

**Table 3:** Submandibular gland MR sialography findings in patient and control group cases.

Submandibular MR sialography findings		
	Diseased cases (*n* = 40)	Control groups (*n* = 20)
Normal	13 (32.5%)	20 (100%)
Chronic sialoadenitis	8 (20%)	—
Ductal ectasia	7 (17.5%)	—
Stone	10 (25%)	—
Sjögren syndrome	2 (5%)	—
